# The Hospital Library and Charities Bureau

**Published:** 1906-12-29

**Authors:** 


### THE HOSPITAL LIBRARY AND CHARITIES BUREAU.

This institution has been founded as a centre of reference in all matters concerning the establishment and management of charities. It is open to all interested in the subject on payment of a small annual fee, and inquiries by members can be made by post. Write for prospectus to the Librarian, The Hospital Buildings, 28 & 29 Southampton Street, Strand.

Inquiries of the Librarian are answered in this column free of charge. Inquiries to be answered promptly by post must be accompanied by a fee of 2s. 6d.

